# The role of three-dimensionality and alveolar pressure in the distribution and amplification of alveolar stresses

**DOI:** 10.1038/s41598-019-45343-4

**Published:** 2019-06-19

**Authors:** Mauricio A. Sarabia-Vallejos, Matias Zuñiga, Daniel E. Hurtado

**Affiliations:** 1 Department of Structural and Geotechnical Engineering, School of Engineering, Pontificia Universidad Católica de Chile, Vicuña Mackenna 4860, Santiago, Chile; 2 Institute for Biological and Medical Engineering, Schools of Engineering, Medicine and Biological Sciences, Pontificia Universidad Católica de Chile, Vicuña Mackenna 4860, Santiago, Chile

**Keywords:** Respiration, Biomedical engineering

## Abstract

Alveolar stresses are fundamental to enable the respiration process in mammalians and have recently gained increasing attention due to their mechanobiological role in the pathogenesis and development of respiratory diseases. Despite the fundamental physiological role of stresses in the alveolar wall, the determination of alveolar stresses remains challenging, and our current knowledge is largely drawn from 2D studies that idealize the alveolar septal wall as a spring or a planar continuum. Here we study the 3D stress distribution in alveolar walls of normal lungs by combining *ex-vivo* micro-computed tomography and 3D finite-element analysis. Our results show that alveolar walls are subject to a fully 3D state of stresses rather than to a pure axial stress state. To understand the contributions of the different components and deformation modes, we decompose the stress tensor field into hydrostatic and deviatoric components, which are associated with isotropic and distortional stresses, respectively. Stress concentrations arise in localized regions of the alveolar microstructure, with magnitudes that can be up to 27 times the applied alveolar pressure. Interestingly, we show that the stress amplification factor strongly depends on the level of alveolar pressure, i.e, stresses do not scale proportional to the applied alveolar pressure. In addition, we show that 2D techniques to assess alveolar stresses consistently overestimate the stress magnitude in alveolar walls, particularly for lungs under high transpulmonary pressure. These findings take particular relevance in the study of stress-induced remodeling of the emphysematous lung and in ventilator-induced lung injury, where the relation between transpulmonary pressure and alveolar wall stress is key to understand mechanotransduction processes in pneumocytes.

## Introduction

The biomechanical behavior of pulmonary alveoli, the basic ventilatory unit of the lung, plays a fundamental role in the respiratory physiology of mammalians. Comprising over 70% of the lung volume, alveoli are constantly stretched by parenchymal internal stresses to accommodate the inspired air, to later expel air out of the lung by elastic recoil during deflation^1^. During such ventilatory cycle, alveoli undergo dynamic states of stress, defined as the force acting on a unit area of tissue, as well as strain, which is a measurement of local deformation of the tissue. These mechanical changes at the level of the extracelullar matrix have long intrigued lung physiologists and pulmonologists, as they dictate the overall lung mechanical behavior^2^. More recently, the determination of stresses in the alveolar septal wall has gained great relevance due to the mechanobiological role of stresses in lung remodeling and pathogenesis, particularly in the case of pulmonary emphysema^3^. A pioneering study in the field of lung mechanics is the work by Mead and collaborators^4^, where fundamental mechanical principles are enforced in a simplified representation of the lung parenchymal tissue to analytically explain how alveolar volume and stresses are related to transpulmonary pressure and tidal volume. An important conclusion was that lung inhomogeneity, interpreted as abrupt changes in the configuration and internal pressure of one alveolar unit embedded in an otherwise uniform parenchymal structure, leads to a stress amplification in the walls of the neighboring units. The concepts of lung inhomogeneity and the associated local stress amplification have recently attracted great attention by the intensive-care medicine community, as the existence of highly localized levels of stress in alveoli is currently considered as one of the main determinants of ventilator-induced lung injury^5,6^. Further, there is wide clinical consensus that organ-level deformation measures are not discriminant enough to detect the early-stage onset of harmful pulmonary conditions during mechanical ventilation therapy^7^, which has motivated the development of novel regional tissue biomarkers that can better represent the effect of mechanical ventilation on the strains and stresses acting on lung tissue^8,9^.

The determination of stresses in alveolar walls remains an outstanding technological challenge. Current methods rely on the mechanical analysis of *ex-vivo* images of the lung parenchyma. Based on scanning electron microscopy images, Gefen *et al*.^10,11^ developed a finite-element model of a sample of murine lung tissue, whose underlying geometry was constructed from micrographs of alveolar sacs with enough resolution to resolve key histological features. By imposing static equilibrium in the continuum domain representing the alveolar sac under different levels of alveolar pressure, they were able to resolve the deformed state and the stress distribution in the sample, concluding that local stresses can concentrate in localized regions of the parenchyma, a phenomenon that is amplified in emphysematic lungs. Dynamic images of isolated perfused rat lungs have also been used to study the relation between transpulmonary pressure and alveolar perimeter distension by using confocal fluorescence microscopy^12,13^. Based on a similar imaging technique, estimates of septal stress, strain and elastic modulus were obtained for subpleural regions of the lung^14^. To this end, the alveolar sacs were idealized as a network of non-linear springs representing the alveolar septum. One important limitation shared by most investigations to date is the two-dimensional (2D) nature of the acquired images, which neglects the out-of-plane geometry and mechanics of the alveolar sacs. As a consequence, the associated mechanical analysis requires the assumption of out-of-plane conditions, either of the plain-stress or plain-strain type, neither of which are truly representative of the stress state in the lung parenchyma, which is inherently three-dimensional (3D). Another key shortcoming is the use of spring (axial) elements to model the alveolar septa. By definition, spring elements can only carry uniform uniaxial stresses, neglecting cross-section gradients in the normal stresses, as well as the transversal stress arising from the application of the alveolar pressure on the surface of alveolar walls, thus limiting the accuracy of the stress analysis.

Micro-computed tomography (*μ*-CT) has enabled the morphological study and volumetric reconstruction of the acinar architecture with high fidelity in a less invasive way than traditional histological techniques^15,16^. The possibility of reconstructing 3D images of the lung with micrometer resolution has motivated a number of morphometric studies, where the lung parenchyma of rats has been characterized in terms of airspace volume, equivalent diameter and surface-to-volume ratio, enhancing and correcting the previous knowledge purely based on 2D histological analyses^17,18^. Spatial 3D representations of the pulmonary acinus constructed from *μ*-CT have also been reported in the literature, where tetrahedral meshes have been constructed from *μ*-CT images to visualize and study the intricate alveolated 3D architecture of acini^19^. Such geometrical representation of the acinus has motivated the development of structural models of the lung parenchyma by means of 3D continuum finite-element (FE) simulations^20–22^, which have allowed for a detailed account of the distribution of local strains in the alveolar walls of a rat acinus sample. These *in-silico* experiments prescribed a uniform displacement field on the system boundary that was associated to arbitrary uniaxial elongation and simple shear deformation applied to the domain boundary, to determine, in a continuum fashion, the strain distribution in the alveolar walls of the sample under simplified loading conditions. One key conclusion from these 3D numerical simulations is that local strain can be highly heterogeneous in the alveolar-wall sector, which confirms the conclusions reached by previous 2D *in-silico* studies. In particular, strain concentrations with amplitudes up to four times the average strain were found in the analyzed samples^20^. While these findings provide key insights on the 3D deformation of acinus under idealized boundary conditions, an accurate and complete study of the 3D stress distribution in the alveolar walls of acinus under realistic loading conditions remains open.

In this work, we thoroughly assess the fully 3D stress distribution in the alveolar walls of normal rat acini by determining the stress tensor field. We hypothesize that the consideration of 3D alveolar geometries and the use of continuum stress analysis on deformed configurations deliver 3D distributions of stresses that will enhance our current knowledge of the distribution and amplification of alveolar stresses, established predominantly from previous 2D studies. To this end, we develop a combined experimental-computational technique to assess the 3D stress distribution in alveolar walls of rat acini. We then characterize our results in statistical terms, and contrast them to current alveolar stress states reported in the literature.

## Results

**Figure 1:**
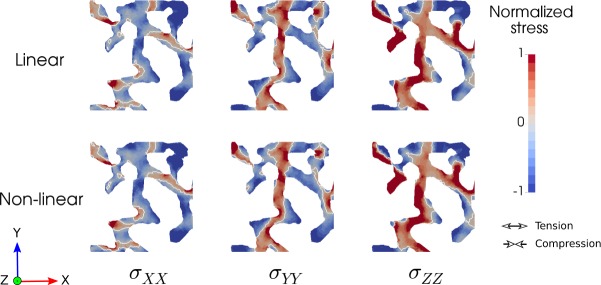
Normalized stress component fields in a section of a representative HAP sample (20 cm H_2_O) for the linear and non-linear analyses. Regions of stress concentrations are observed throughout the samples, showing the high dispersion of stress levels.

Morphometric analysis resulted in a surface-to-volume ratio of 94.48 ± 6.98 mm^−1^ for LAP group, and 76.42 ± 6.24 mm^−1^ for the HAP group. The porosity was 0.63 ± 0.02 and 0.7 ± 0.01 for the LAP and HAP groups, respectively (Tables Sup. 1 and 2, Supplementary material). The distribution of normalized stress components *σ*_*XX*_, *σ*_*YY*_ and *σ*_*ZZ*_ for a plane section of a representative sample of the HAP group is shown in Fig. 1 for the linear and non-linear analyses. No appreciable differences are found between the stress distributions arising from linear and non-linear analysis. In all stress components, the spatial distribution is highly heterogeneous, and stress concentrations are found in localized regions of the alveolar domain. Interestingly, the *σ*_*XX*_ field shows positive values, which are associated to tension in the *X*−direction, in elongated regions that are aligned with the *X*−direction. Further, negative values of *σ*_*XX*_ are found in elongated regions aligned with the *Y*−direction, i.e., orthogonal to the stress-component direction. An analogous trend is observed for the *σ*_*YY*_ field.

The mesh-quality analysis showed that, in all the meshes generated, 99% of the elements had a Joe-Liu parameter greater than 0.51 (Figure Sup. 1(a), Supplementary material). The accuracy of the computational stress analysis was assessed by means of a numerical convergence study, which showed no appreciable differences in the von Mises stress distribution for meshes with approximately 750,000 elements when compared to a baseline fine-mesh simulation with 1,200,000 elements (Figure Sup. 1(b), Supplementary material). The stress distribution was found to be highly independent of the value of the elastic constants, as the von Mises stress frequency distribution did not change when increasing the Lamé constant by a factor of ten (Figure Sup. 1(c), Supplementary material). Changes in the disposition of boundary conditions did not significantly affect the stress distribution (Figure Sup. 1(d), Supplementary material). Three RVE sizes were tested, edge size L = 100, 250, and 500 *μ*m, resulting in normalized von Mises distributions with mean values of 6.89, 7.08 and 8.87, respectively. No appreciable differences were found between the cases *L* = 100 *μ*m and *L* = 250 *μ*m. The case with *L* = 500 *μ*m displayed a marked positive shift of the mean value, which also resulted in a longer tail when compared to the smaller edge-size cases. The 95th percentile values were 16.18, 16.23 and 27.51 for the L = 100, 250, and 500 *μ*m cases, respectively, which supports the observation of a larger tail in the distribution of the largest RVE.

**Figure 2:**
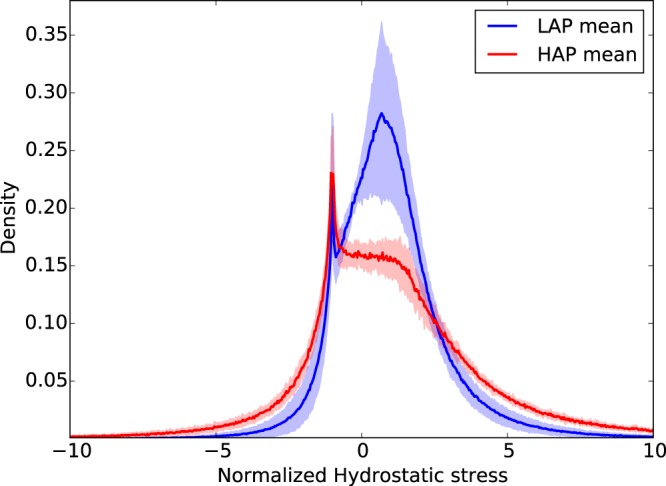
Frequency distribution of normalized hydrostatic stress for LAP and HAP group: Solid line shows the group average distribution, the lighter envelope shows the standard deviation of the group distributions. A bimodal shape is observed for the LAP group, while the positive peak is smeared in the HAP group.

**Figure 3:**
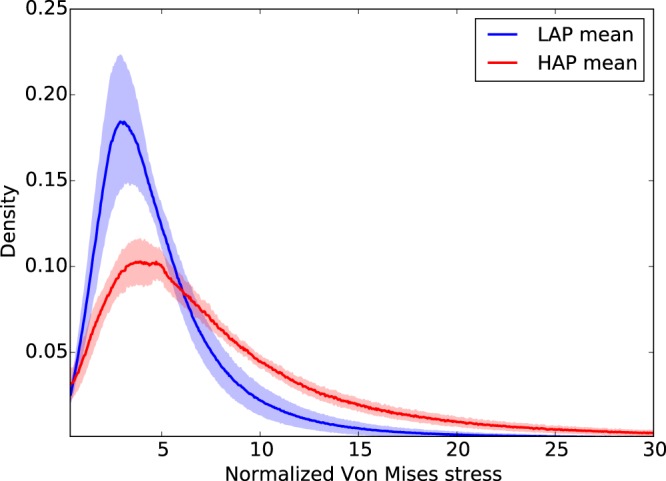
Frequency distribution of normalized Von Mises stress for LAP and HAP group: Solid line shows the group average distribution, the lighter envelope shows the standard deviation of the group distributions. Unimodal and positively skewed distributions were observed in both groups, with the HAP group displaying a higher dispersion in stresses than the LAP group.

The frequency distribution of the normalized hydrostatic stress in the LAP group displays a marked bimodal trend with peak values in the negative (−1.0) and positive (+0.67) ranges (Fig. 2). The HAP group displays a clear peak on the negative range (−1.0) which coincides with the negative peak found in the LAP group (Fig. 2). Frequency distributions for the von Mises stress in the LAP and HAP groups are reported in Fig. 3. Clear unimodal and positively skewed distributions are observed for both groups. However, the LAP group displays a more concentrated and less skewed distribution than the HAP group which displays a heavier right tail.

**Figure 4:**
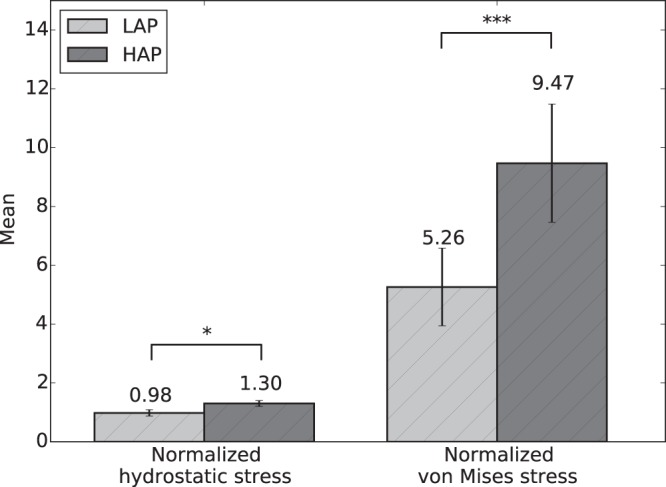
The effect of alveolar pressure on stress amplification: Comparison of group mean values of normalized hydrostatic and von Mises stresses between the LAP and HAP groups. Significant differences between groups were found for both stress measures studied. (*p ≤ 0.05, **p ≤ 0.01, and ***p ≤ 0.001).

To describe the heterogeneity of the stress measures in quantitative terms, the mean, mode, 5^*th*^ and 95^*th*^ percentiles of normalized hydrostatic and von Mises stress measures for the LAP and HAP are reported in Table Sup. 1 (Supplementary material) and Table Sup. 2 (Supplementary material), respectively. We note that in the LAP group, the 95^*th*^ percentiles for the hydrostatic stress and von Mises stress were 4.5× and 12.6× the alveolar pressure, confirming that high stress concentrations do arise in small regions of the RVEs. This effect is also observed in the HAP group, where the 95^*th*^ percentiles for the hydrostatic stress and von Mises stress were 8.4× and 26.9× the alveolar pressure. A comparison of group mean values of normalized hydrostatic stress and normalized von Mises stress can be found in Fig. 4. For both stress measures, the normalized stress values were found to be significantly different between the LAP and HAP groups.

**Figure 5:**
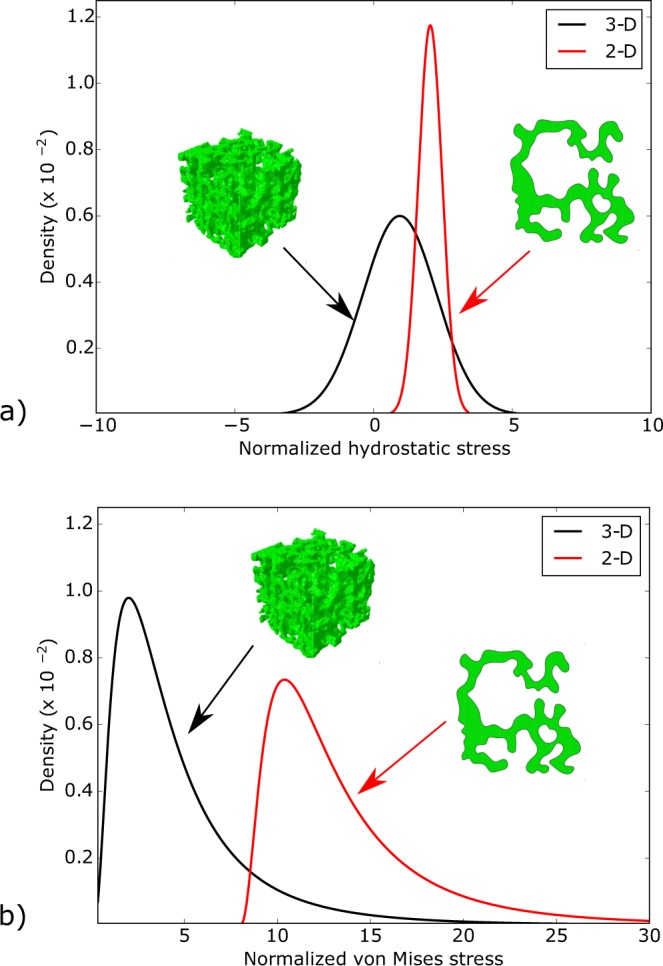
Difference between 2D and 3D analyses of alveolar stresses: Stress distributions for (**a**) normalized hydrostatic stress distribution, and (**b**) normalized von Mises stress distribution. 2D stress analysis consistently results in distributions with higher values than those obtained from 3D stress analysis, suggesting an overestimation of 2D methods.

**Figure 6:**
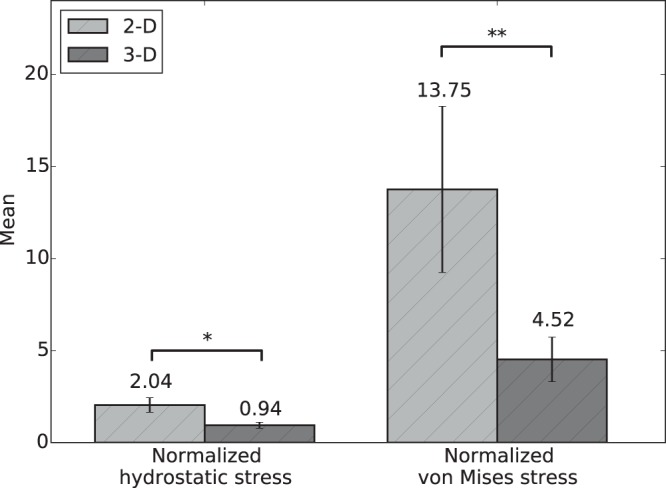
Difference in mean alveolar stresses between 2D and 3D analyses: Significant differences are found in alveolar stresses determined from 2D and 3D methods for both stress measures studied (*p ≤ 0.05, and **p ≤ 0.01).

Alveolar stresses resulting from 3D stress analysis were significantly different from those obtained from 2D analysis. The 2D normalized hydrostatic stress distribution was found to have a higher mode and more concentrated distribution than its 3D counterpart (Fig. 5(a)). The distribution of von Mises stresses for the 2D case also resulted in a mode higher than that observed in the 3D case. Mean values for both stress measures were found to be significantly different between the 2D and 3D case (Fig. 6).

## Discussion

In this work, we have assessed the 3D stress distribution in alveolar walls of rat lungs under high and low alveolar-pressure levels. While the estimation of alveolar strains and stresses has been the focus of several investigations in lung mechanics in the past^10,20,21^, the present work represents a first attempt to characterize alveolar stresses in statistical terms using realistic three–dimensional alveolar architectures under the effect of different levels of alveolar pressure.

One novel aspect of our work is the consideration of the fully 3D stress state in the alveolar wall, which is subject to realistic loading conditions acting on deformed configurations at different alveolar pressure levels. First, we note that the resulting stress tensor distributions do not significantly change when considering a non-linear constitutive relation, which supports our approach of using a linear elasticity model. Further, the stress distributions do not seem to be affected by global changes in the elastic modulus, neither by the disposition of the boundary conditions. These findings suggest that the stress tensor field in alveolar architectures is predominantly statically determinate, i.e., the stress tensor field largely depends on satisfying the differential equilibrium equations. Another remarkable observation is that the stress component along the long axis of elongated subregions in the RVE tend to be under tension, while stress components orthogonal to this axis tend to be under compression, see Fig. 1. Further, the magnitudes of tensile and compressive stresses are similar in range. Thus, we conclude that alveolar walls are subject to a multiaxial 3D stress state, with non-negligible levels of stress acting in directions that are orthogonal to the main direction of the tissue. This finding supports the need of fully 3D analyses when characterizing alveolar stresses, an approach that largely departs from the traditional consideration of the acinus as a network of spring (1D) elements that only carry axial stress^4,23–25^.

To facilitate the analysis of the distribution of stress in acinar geometries, we propose considering the hydrostatic and von-Mises stress measures of the stress tensor field, which correspond to dilatational and distortional deformation mechanisms, respectively. These stress measures have the advantage of being scalars that are invariant to changes of reference frames, and whose distribution in space can be analyzed in statistical terms^26^. Our results show that the frequency distributions of hydrostatic and von-Mises stresses cannot be represented by a normal distribution, highlighting the importance of characterizing statistical parameters other than the mean and standard deviation. Interestingly, we note that the shape of normalized stress frequency distributions depends on the applied alveolar pressure, as we observe that alveolar stresses in the HAP group result in distributions with heavier tails than those in the LAP group, i.e., an increase of the applied alveolar pressure results in larger spread in the stress magnitudes (Figs 2 and 3). This effect is further evidenced by the von Mises stress distributions when comparing the 95^*th*^ percentile of the normalized stress: 12.6× the applied alveolar pressure in the LAP group versus 26.9× in the HAP group. This dependence on the level of applied alveolar pressure also arises when comparing normalized mean stress magnitudes, both for hydrostatic and von Mises measures, which are in all cases significantly different (Fig. 4). Therefore, we conclude that stress amplification in the alveolar wall cannot be characterized by a unique amplification factor, but should be related to the level of applied alveolar stress. This takes particular relevance in lung physiology, where the concept of stress raisers in the lung tissue has been represented by an amplification factor that is independent of the level of transpulmonary pressure^4,6,25,27^. An interesting avenue of research is to investigate potential changes in the composition of the underlying structural components that bear such high stress concentrations^28^, as well as unveiling how stress concentrations drive remodelling processes in the extracellular matrix^2^. Further, it is important to remark that the predominant structure carrying loads in the alveolar walls is the fiber network composed by elastin and collagen fibers embedded in the extracellular matrix^29^. Despite its relevance, the specific forces being carried by each of these structural components remains largely unknown. Future contributions should focus on how alveolar stress is resisted by these structures, which are known to protect the delicate blood-gas barrier formed by epithelial and endothelial cells, among other elements^30^.

Another novel conclusion of this work is that 2D mechanical analysis of acinar architectures tends to overestimate the stress level in alveolar walls. A direct comparison of the stress frequency distribution resulting from 2D and 3D evidences a positive shift towards higher values both for the hydrostatic and von Mises stresses (Fig. 5), with mean values for the 2D case that are significantly larger that their 3D counterparts (Fig. 6). The tendency to overestimate observed in 2D analysis can be attributed to the omission of out-of-plane stresses, which contribute to bearing the applied alveolar pressure and internal stresses mainly by membrane stresses, and thus they carry part of the total loading, resulting in a reduction of stresses for the in-plane stress components. This effect is readily seen when comparing the stresses generated by an internal pressure *p* acting on a thin ring (2D structure) and those arising in a thin hollow sphere (3D structure) under the same internal pressure. In the former, the resulting membrane stress is ${\sigma }_{{\rm{ring}}}=\frac{pR}{e}$, whereas in the latter ${\sigma }_{{\rm{sphere}}}=0.5\frac{pR}{e}$, where *R* is the radius and *e* is the membrane thickness, from which we obtain that *σ*_ring_ = 2 × *σ*_sphere_, i.e., a 2D analysis delivers a stress level that is twice the stress present in the 3D structure.

To further put our findings in perspective with previous results, we assume the case of an axial element under principal stresses, subject to axial stress *σ*_ax_ along the longitudinal direction, and to alveolar pressure *p*_alv_ in the transversal plane, see Figure Sup. 2. From the definition of hydrostatic stress (6), we can rewrite the axial stress as

1 $${\sigma }_{{\rm{ax}}}=3{\sigma }_{H}+2{P}_{alv}$$

**Figure 7:**
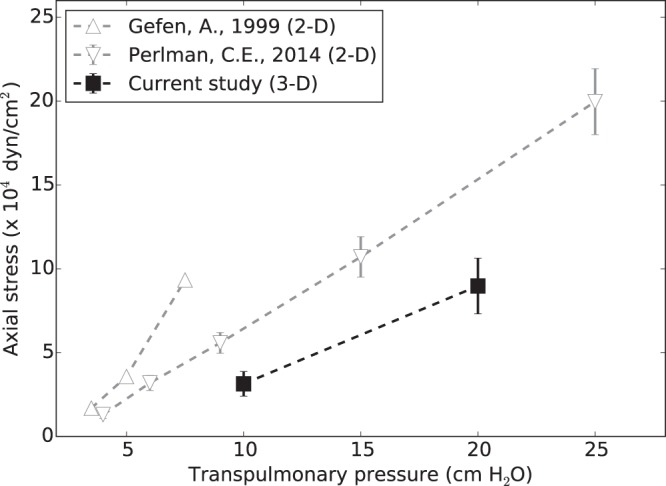
Axial stress in alveolar walls: Comparison of equivalent axial stresses computed from 3D analysis versus axial stresses determined from 2D methods reported in the literature.

Using (1) we estimate an equivalent axial stress from the distribution of hydrostatic stresses. We further note that in our case, lungs were scanned after being removed from the thorax cavity, which implies that, due to the absence of intrapleural pressure, the transpulmonary pressure (P_tp_) is the same as the alveolar pressure (P_alv_). Figure 7 shows the axial stress versus transpulmonary pressure obtained from this study, along with the results reported from 2D studies of alveolar stress^10,14^. Once again, we observe that both 2D studies report stresses that are higher than those obtained from our 3D study, which is in line with our findings. Therefore, we conclude that the estimation of alveolar stresses is strongly influenced by the dimensionality assumed by the method of analysis, and that the explicit consideration of the out-of-plane dimension will result in lower levels of alveolar stress, which can be considered closer to the actual stresses due to the explicit consideration of the three dimensionality of the alveolar architecture. We note here that the results of Perlman and Bhattacharya^14^ correspond to air-filled subpleural alveolar structures, which poses important differences in the experimental setup and in the region inside the lung analyzed when compared to our methodology. While we expect that stress distributions will display variations depending on the position inside the lung and on the experimental settings, our study confirms a significant reduction of stress levels when the third dimension is explicitly considered in the mechanical analysis. This finding is paramount to future studies of lung disease mechanobiology, as stresses in the alveolar wall are believed to trigger remodeling processes in the lung tissue that give origin to respiratory diseases such as emphysema^3,31^.

Our work suffers from some limitations which we discuss next. During the construction of the finite-element models the mechanical effect of the surface tension induced by the alveolar fluid was not included. Depending on the level of tidal volume, surface tension can result in additional stresses acting on the interalveolar septa^32^, a mechanical contribution that markedly impacts the global mechanics of the lung^33^. During normal breathing, variations of surface tension can be small, and may not exceed a range of 5 mN/m. However, large volume changes induce large changes in the alveolar surface which results in non-negligible variations in the surface tension, and therefore in the stresses that arise in the alveolar walls to resist surface tension^30^. Based on these observations, we remark that the stress distributions obtained in this work can be assumed to be accurate for the zero surface-tension limit, but necessitate corrections when alveolar surfactant is present^34^. Future contributions should incorporate the effect of surface tension, which needs accurate information not only of the alveolar geometry and alveolar pressure but also of the surfactant concentration and adsorption-desorption kinetics^35^, which are currently challenging to assess *in-situ*. Another limitation of the methodology is the formalin-based procedure employed for fixation, which is known to cause shrinkage and incomplete fixation of elastic fibers^36^. A parameter commonly used in assessing shrinkage of lung tissue samples is the alveolar surface-to-volume ratio, which in mice has been found to be in the range of 50–100 mm^−1^ for the C57Bl/6 J mouse^18,37,38^. The values obtained in this study fall in that range, as the average surface-to-volume ratios were 94.48 and 76.42 for the LAP and HAP groups, respectively.

## Methods

### *μ*-CT imaging of rat lungs

The study protocol was approved by the Bioethics Committee of the Pontificia Universidad Católica de Chile. Healthy adult male Sprague-Dawley rats (256 ± 16 gr, N = 6) were considered for this study. Animals were sacrificed according to the international guidelines given by the American Veterinary Medical Association (AVMA)^39^. A tracheostomy procedure was performed to control lung formalin phosphate-buffered-saline (F-PBS) flow by means of a fixed cannula with a three-step valve to prevent fluid leaking. Animals were randomly allocated to either at LAP group (N = 3) or at HAP group (N = 3). The F-PBS solution was slowly pumped through the cannula into the lungs until reaching the target airway pressure measured through a syringe with a pressure transducer (AG Cuffil, Hospitech Respiration Ltd.). Airway pressure was manually adjusted to target values and monitored for ten minutes, after which variations in airway pressure were below 0.1 cm H_2_O within a time frame of 5 minutes. The animals were refrigerated overnight maintaining the airway pressure constant by carefully stitching the trachea. After this first F-PBS fixation stage, lungs with trachea and cannula were carefully excised from the rat thoracic cavity, to then submerge them into a F-PBS solution for 24 hours^17^. Then, the left lung was dissected and dried by following a desiccating process based on gradual ethanol baths^40^. Lastly, lungs were maintained in a 100% ethanol bath overnight^41,42^. Previous to the scanning process, left lungs were left air drying at atmospheric conditions during 24 hours in order to evaporate the remaining ethanol.

**Figure 8:**
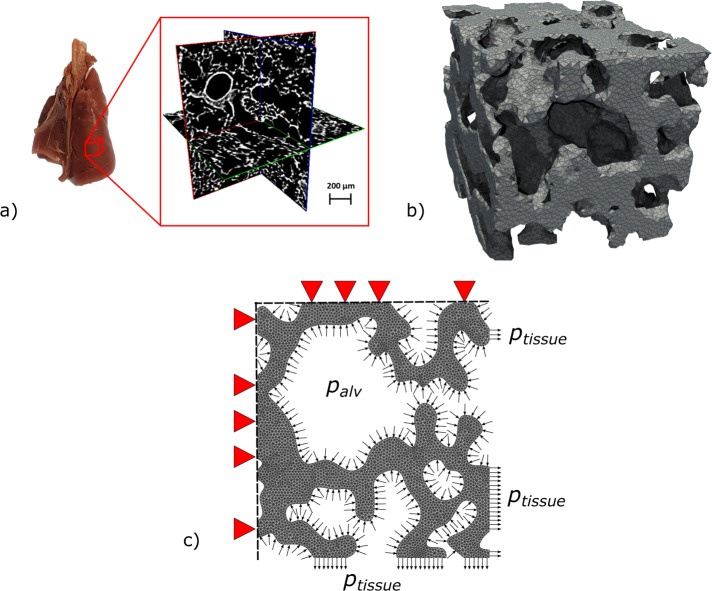
Generation of the computational model to assess 3D alveolar stresses. (**a**) Cuboid subdomains are selected from *μ*-CT whole-lung images to define the geometry of an RVE, avoiding larger airways in the sample to obtain mostly acinar tissue, (**b**) finite-element meshes are generated from acinar images to represent the alveolar architecture, (**c**) boundary conditions include alveolar pressures acting on the internal surfaces, displacement prescription on three bounding faces of the RVE, and orthogonal stress representing the tissue reaction acting on the remaining bounding faces of the RVE.

Fixed lungs were scanned using a commercial *μ*-CT device (SkyScan 1272, Bruker). The X-ray source voltage and current were set to 10 kV and 250 *μ*A, respectively. Whole-lung tomographic scans were acquired using an isotropic voxel resolution of 2.7 *μ*m per voxel. 3D images of the lung were reconstructed using the NRecon software (Bruker), where misalignment compensation, ring artifact reduction and hardening filters were employed to improve the image quality. 3D images were segmented using a multi-level thresholding procedure based on Otsu method to isolate the lung parenchyma from surrounding organs, major vessels and airways^43,44^. These values were in concordance with common tissue Hounsfield intensities. See Fig. 8(a) for a representative *μ*-CT image.

Alveolar cubic samples, also termed here as representative volume elements (RVE), with an edge size of 250 *μ*m, were selected from image reconstructions of the left lung, resulting in 6 RVEs for the low alveolar pressure group (LAP, airway pressure of 10 cm H_2_O) and 6 RVEs for the high alveolar pressure group (HAP, airway pressure of 20 cm H_2_O). RVEs in the same group were chosen so as to have similar morphological parameters such as mean alveolar radius, surface-to-volume ratio, and porosity^18,38^. Care was exercised in excluding regions that included portions of large bronchi, as well as avoiding zones close to the pleura and close to larger airways. Figure 8(b) shows one representative RVE. Porosity, defined as the ratio between the air volume over the total volume of an RVE (including air and alveolar tissue) was assessed from the reconstructed masked images.

### 3D alveolar stress analysis

To determine the stress distribution in the alveolar walls, we considered an elasticity formulation for the alveolar tissue based on the geometry obtained from *μ*-CT images. It is important to remark that alveolar stresses are in equilibrium in the deformed configuration, which in our case is given by the acquired images. Therefore, we followed a spatial formulation of the elasticity problem, *i.e*., the analysis was based on the deformed configuration of the tissue, which we denote by Ω. Under equilibrium, the 3D Cauchy stress tensor field *σ* is governed by the differential equilibrium equation

2 $${\rm{div}}\,\sigma =0\,{\rm{in}}\,{\rm{\Omega }},$$

We denote by *u* the displacement field associated to the equilibrium configuration of the alveolar domain. To reduce the computational complexity of the finite-element model, we assumed a linear elastic response for the alveolar tissue. Further, we assumed the alveolar tissue maintains its volume, i.e., it behaves as an incompressible material. Under these assumptions, the constitutive law takes the form.

3 $$\sigma =-\,p{I}+2\mu \varepsilon ({u}),$$

where *μ* is the elastic Lamé constant, *p* is the static pressure field, *I* is the identity tensor, and the infinitesimal strain tensor is defined as

4 $$\varepsilon ({u})\,:\,=\frac{1}{2}\{\nabla {u}+\nabla {{u}}^{T}\}.$$

We set the elastic modulus to *E* = 9.5 × 10^5^ dyn/cm^2^ following values reported in the literature^24^ and the Poisson ratio to *ν* = 0.5, as we assume the extracellular matrix maintains its volume (incompressible material), which yields $\mu =\frac{E}{2(1+\nu )}=3.17\times {10}^{5}\,{\rm{dyn}}/{{\rm{cm}}}^{2}$. To assess the accuracy of the linear elastic stress analysis, we also performed a non-linear finite-element analysis for one RVE case, and compared the resulting stress distributions with those obtained from linear-elastic analysis. To this end, we followed a non-linear hyperelastic formulation that allows for large deformations, and assumed that the alveolar walls behaved as an incompressible Neo-Hookean material^20^.

Appealing to Pascal’s law for incompressible fluids, and neglecting the effect of gravity, alveolar pressure (*p*_*alv*_) was assumed to be equal to the pressure imposed at the airways in the experiments. Since lungs are scanned after being removed from the thoracic cage, intrapleural pressure is absent, and transpulmonary pressure can be assumed to be equal to the alveolar pressure. Alveolar pressure was applied to all surfaces exposed to air inside each RVE. Displacement boundary conditions were enforced in three orthogonal faces where zero normal displacements were prescribed, while the remaining three bounding faces were subject to tractions representing the effect of the sorrounding tissue, see Fig. 8c). To determine these tractions, we considered the net force equilibrium on each of the three bounding faces in the RVE independently. For a particular traction bounding face, let *A*_*tissue*_ be area occupied by the intersection of the alveolar tissue with the bounding face, *p*_*tissue*_ be the pressure acting such tissue area representing the tractions exerted by the sorrounding tissue, and *A*_*air*_ be the area of the boundary face intersected by airspace under alveolar pressure. Force balance on the bounding face implies that

5 $${p}_{tissue}={p}_{alv}\frac{{A}_{air}}{{A}_{tissue}}.$$

Displacement boundary conditions were enforced in three orthogonal faces where zero normal displacements were prescribed, and uniform pressure with magnitude *p*_*tissue*_ was set as the boundary condition on each opposite face in the outward direction.

Finite-element models were constructed using the software ABAQUS (Abaqus/CAE 2017, Dassault Systems Simulia Corp., Johnston, RI, USA). To this end, we first generated 3D tetrahedral meshes from the alveolar wall domain segmented in each RVE using the CGAL library^45^. The incompressible elasticity problem was approach using a mixed formulation, where *P*_2_ − *P*_1_ Taylor-Hood tetrahedral (quadratic) elements were employed. Surface loads and boundary conditions were assigned accordingly. To assess the differences between 3D and 2D stress analyses, we performed a 2D FE simulation of a plane section obtained from a suitable RVE, assuming a plane-stress condition.

The stress distribution in the alveolar walls was studied using two classical scalar stress measures: (i) the hydrostatic stress, defined as the first invariant of the Cauchy tensor,

6 $${\sigma }_{H}\,:\,=\frac{1}{3}{\rm{trace}}(\sigma ),$$

and (ii) the von Misses stress, defined as

7 $${\sigma }_{VM}\,:\,=\sqrt{\frac{3}{2}{\sigma }_{dev}:{\sigma }_{dev}}$$

where: signifies the tensor scalar product, and the deviatoric component of the stress tensor reads

8 $${\sigma }_{dev}\,:=\sigma -{\sigma }_{H}{I}.$$

From definitions (6) and (8) we note that the hydrostatic stress and the Von Mises stress quantify two independent and orthogonal components of the Cauchy stress tensor, i.e, the hydrostatic and deviatoric behavior, respectively. We also note that in isotropic solids, the hydrostatic stress can be related to changes in volume, whereas the deviatoric stress is related to shape changes.

To assess the numerical accuracy of the computational model, a mesh quality analysis based on the Joe-Liu parameter was performed^46^, and histograms were constructed to check that the majority of elements had an acceptable parameter value. A numerical convergence analysis was also performed for one RVE case using a tetrahedral discretization with a total number of elements ranging from 70 k to 1200 k. The resulting stress distributions were contrasted by means of the resulting frequency distributions of the stress measures defined above. To assess the sensitivity of the stress analysis to variations in the elastic properties of the alveolar tissue, we studied the impact on the stress measure frequency distribution in one RVE case using a Lamé constant 10× higher than the standard value adopted. The sensitivity of the stress distributions to the disposition of boundary conditions and to the RVE domain size was also analyzed.

The resulting stress analyses were summarized in terms of relative frequency distributions of the two stress measures considered in this study. Stress measures were normalized by the applied alveolar pressure, to allow for a direct comparison of stress amplification and heterogeneity between experimental groups. Values are expressed as mean ± standard deviation, unless noted otherwise. Significant differences between group mean values were assessed using the Mann-Whitney-Wilcoxon test for non-parametric distributions.

## Supplementary information

Supplementary Material
