# Supplementary material for: The role of three-dimensionality and alveolar pressure in the distribution and amplification of alveolar stresses
Source: Sci Rep. 2019 Jun 19;9:8783. doi: 10.1038/s41598-019-45343-4 (PMC6584652; doi:10.1038/s41598-019-45343-4)
Supplement: Supplementary file 1 — Supplementary Material [file 41598_2019_45343_MOESM1_ESM.pdf]

# The role of three-dimensionality and alveolar pressure in the distribution and amplification of alveolar stresses

**Mauricio A. Sarabia-Vallejos[1,2], Matias Zuñiga[1], and Daniel E. Hurtado[1,2,*]**

[1]Department of Structural and Geotechnical Engineering, School of Engineering, Pontificia Universidad Católica de Chile, Vicuña Mackenna 4860, Santiago, Chile
[2]Institute for Biological and Medical Engineering, Schools of Engineering, Medicine and Biological Sciences, Pontificia Universidad Católica de Chile, Vicuña Mackenna 4860, Santiago, Chile
[*]Corresponding author e-mail: dhurtado@ing.puc.cl

## ABSTRACT

Alveolar stresses are fundamental to enable the respiration process in mammalians and have recently gained increasing attention due to their mechanobiological role in the pathogenesis and development of respiratory diseases. Despite the fundamental physiological role of stresses in the alveolar wall, the determination of alveolar stresses remains challenging, and our current knowledge is largely drawn from 2D studies that idealize the alveolar septal wall as a spring or a planar continuum. Here we study the 3D stress distribution in alveolar walls of normal lungs by combining ex-vivo micro-computed tomography and 3D finite-element analysis. Our results show that alveolar walls are subject to a fully 3D state of stresses rather than to a pure axial stress state. To understand the contributions of the different components and deformation modes, we decompose the stress tensor field into hydrostatic and deviatoric components, which are associated with isotropic and distortional stresses, respectively. Stress concentrations arise in localized regions of the alveolar microstructure, with magnitudes that can be up to 27 times the applied alveolar pressure. Interestingly, we show that the stress amplification factor strongly depends on the level of alveolar pressure, i.e, stresses do not scale proportional to the applied alveolar pressure. In addition, we show that 2D techniques to assess alveolar stresses consistently overestimate the stress magnitude in alveolar walls, particularly for lungs under high transpulmonary pressure. These findings take particular relevance in the study of stress-induced remodeling of the emphysematous lung and in ventilator-induced lung injury, where the relation between transpulmonary pressure and alveolar wall stress is key to understand mechanotransduction processes in pneumocytes.

## Supplementary information

**Table Sup.1.** LAP group ($p_{\text{alv}} = 10$ cm $H_2O$): Statistics for porosity, normalized hydrostatic stress (5%, mode, mean, 95%), and normalized von Mises stress (5%, mode, mean, 95%)

| RVE | Porosity | Normalized Hydrostatic stress | | | | Normalized Von Mises | | | |
|---|---|---|---|---|---|---|---|---|---|
| | | **5 %** | **Mean** | **Mode** | **95 %** | **5 %** | **Mean** | **Mode** | **95 %** |
| 1 | 0.61 | -1.19 | 0.81 | 1.17 | 3.32 | 1.09 | 4.09 | 2.48 | 9.39 |
| 2 | 0.63 | -1.24 | 0.94 | 1.10 | 3.74 | 1.10 | 4.52 | 2.40 | 10.52 |
| 3 | 0.63 | -1.39 | 1.11 | 1.08 | 4.39 | 1.32 | 5.19 | 3.69 | 12.06 |
| 4 | 0.62 | -2.48 | 0.91 | 0.99 | 5.50 | 1.34 | 6.41 | 4.26 | 15.99 |
| 5 | 0.63 | -1.29 | 1.03 | 0.90 | 4.01 | 1.21 | 4.84 | 3.24 | 10.85 |
| 6 | 0.67 | -2.27 | 1.07 | 0.95 | 5.90 | 1.18 | 6.51 | 3.41 | 16.97 |
| **Mean** | 0.63 | -1.64 | 0.98 | 1.03 | 4.48 | 1.21 | 5.26 | 3.25 | 12.63 |
| **Std.** | 0.02 | 0.57 | 0.11 | 0.10 | 1.02 | 0.11 | 1.00 | 0.71 | 3.12 |

**Table Sup.2.** HAP group ($p_{\mathrm{alv}} = 20$ cm $H_2O$): Statistics for porosity, normalized hydrostatic stress (5%, mode, mean, 95%), and normalized von Mises stress (5%, mode, mean, 95%).

| **RVE** | **Porosity** | **Normalized Hydrostatic stress** | | | | **Normalized Von Mises** | | | |
|---|---|---|---|---|---|---|---|---|---|
| | | **5 %** | **Mean** | **Mode** | **95 %** | **5 %** | **Mean** | **Mode** | **95 %** |
| 1 | 0.71 | -3.62 | 1.29 | -0.96 | 8.59 | 0.82 | 8.96 | 6.49 | 24.90 |
| 2 | 0.71 | -5.22 | 1.39 | -0.92 | 10.59 | 1.46 | 12.13 | 5.38 | 38.33 |
| 3 | 0.71 | -4.76 | 1.11 | -0.89 | 9.05 | 1.11 | 10.89 | 5.15 | 32.04 |
| 4 | 0.71 | -3.15 | 1.63 | -0.92 | 8.62 | 1.49 | 9.21 | 5.27 | 24.46 |
| 5 | 0.69 | -3.08 | 1.15 | -0.96 | 7.23 | 0.98 | 8.19 | 5.18 | 22.88 |
| 6 | 0.70 | -2.39 | 1.23 | -0.94 | 6.47 | 1.02 | 7.41 | 5.28 | 18.68 |
| **Mean** | 0.70 | -3.70 | 1.30 | -0.93 | 8.43 | 1.15 | 9.47 | 5.46 | 26.88 |
| **Std.** | 0.01 | 1.08 | 0.19 | 0.03 | 1.44 | 0.27 | 1.75 | 0.51 | 7.08 |

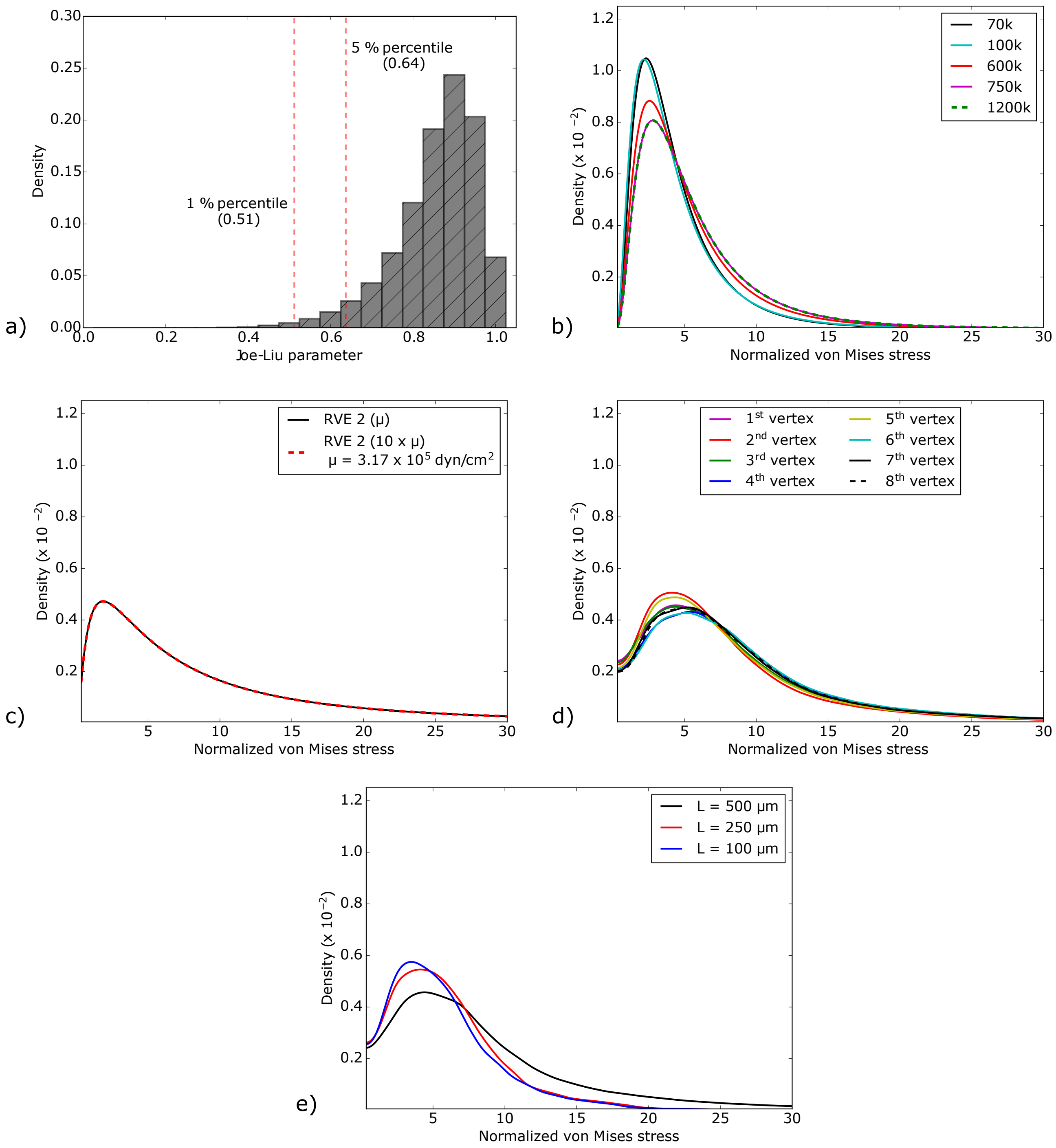


**Figure Sup.1.** Convergence and sensitivity analyses: a) Mesh-quality analysis based on Joe-Liu parameter histogram, b) Mesh convergence analysis, c) Sensitivity of stress distribution to strong variations in the elastic modulus, d) Sensitivity of the stress distribution to changes in boundary conditions, e) Sensitivity of the stress distribution to RVE domain size.

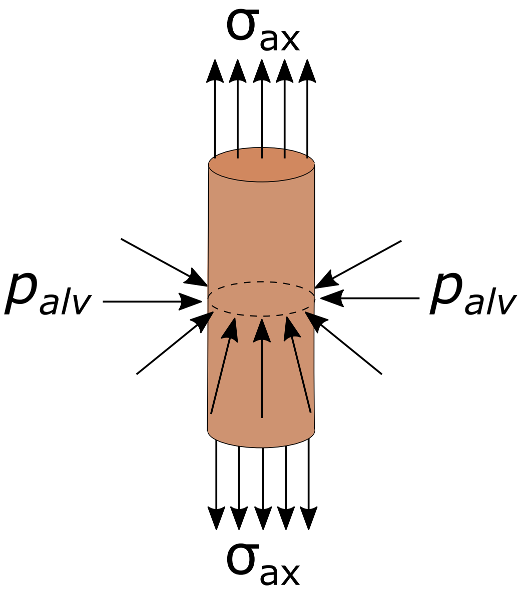


**Figure Sup.2.** Idealization of alveolar wall under multiaxial stress state.
